# Upregulation of HMGB1 in tumor-associated macrophages induced by tumor cell-derived lactate further promotes colorectal cancer progression

**DOI:** 10.1186/s12967-023-03918-w

**Published:** 2023-01-28

**Authors:** Xinyi Gao, Shiqi Zhou, Zhaofu Qin, Dechuan Li, Yuping Zhu, Dening Ma

**Affiliations:** 1 Department of Radiology, The Cancer Hospital of the University of Chinese Academy of Sciences (Zhejiang Cancer Hospital), 1 Banshan East Road, Hangzhou, 310022 Zhejiang People’s Republic of China; 2 Institute of Basic Medicine and Cancer (IBMC), Chinese Academy of Sciences, 1 Banshan East Road, Hangzhou, 310022 Zhejiang People’s Republic of China; 3 Department of Colorectal Surgery, The Cancer Hospital of the University of Chinese Academy of Sciences (Zhejiang Cancer Hospital), 1 Banshan East Road, Hangzhou, 310022 Zhejiang People’s Republic of China; 4 Key Laboratory of Prevention, Diagnosis and Therapy of Upper Gastrointestinal Cancer of Zhejiang Province, Hangzhou, 310022 China

**Keywords:** Colorectal cancer, Tumor-associated macrophages, HMGB1, Lactate

## Abstract

**Background:**

Lactate accumulation leads to an acidic tumor microenvironment (TME), in turn promoting colorectal cancer (CRC) progression. Tumor-associated macrophages (TAMs) are the predominant cells in TME. This study aimed to reveal the regulation mechanism of CRC cell-derived lactate on TAMs and explore the mechanism underlying lactate accumulation-induced aggravation in CRC.

**Methods:**

Cell growth and metastasis were evaluated by colony formation, Transwell, and wound healing assays. Western blot and RT-qPCR were applied to determine the protein and mRNA expression. Flow cytometry was used to analyze the polarization state and apoptotic rate of macrophages induced in THP-1 cells. The lactate in the cell supernatant was quantified using an ELISA kit. Immunofluorescence was performed to visualize the location of High Mobility Group Box 1 (HMGB1). H&E and Ki67 staining assays were used to assess tumorigenesis in nude mice bearing ectopic tumors.

**Results:**

Cell growth and metastasis were promoted in the hypoxic CRC cells. The hypoxic cell supernatant stimulated the M2-type polarization of macrophages. The lactate level increased in hypoxic cancer cells. However, the inhibition of lactate using 3-hydroxy-butyrate (3-OBA) reversed the effects of hypoxia. Also, macrophages showed no promoting effect on cancer cell growth and migration in the presence of 3-OBA. HMGB1 was secreted into the extracellular space of lactate-induced macrophages, further enhancing the malignant behaviors of cancer cells. ERK, EMT, and Wnt signaling pathways were activated in cancer cells due to HMGB1 upregulation.

**Conclusions:**

The lactate metabolized by cancer cells stimulated M2 polarization and HMGB1 secretion by macrophages, aggravating the carcinogenic behaviors of cancer cells.

## Background

Colorectal cancer (CRC) is currently one of the most common malignant tumors in the digestive system, with its mortality rate ranking fourth in the world [1]. CRC develops with the transformation of the normal epithelial mucosa into a highly proliferative epithelium. These highly proliferative intestinal epithelial cells lose their organization and structure, forming adenomas, which can grow and invade the submucosa, become cancerous, and thus invade the colon and metastatic organs [2]. Up to 90% of CRCs are sporadic, implying that the incidence of CRC is closely related to poor lifestyle habits [3]. Patients with immune-and metabolic-related diseases, such as inflammatory bowel disease, obesity, and type 2 diabetes, are also at high risk of CRC [4]. A large body of research evidence suggests that screening asymptomatic individuals at average risk reduces mortality by detecting tumors early and in the curative stage. In addition, certain screening tests can detect precancerous lesions, allowing for earlier removal and lower incidence. Although most patients with early-stage CRC can achieve curative resection or remission through surgery, many patients are still diagnosed with CRC in the middle or advanced stage. The treatment results of CRC are not favorable at present [5]. The current exploration of emerging approaches targeting the tumor microenvironment and some immunotherapies have been shown to be effective in patients with metastatic or intermediate-advanced CRC, which may be beneficial to achieve long-lasting remissions in some patients [6]. Therefore, exploring the potential molecular markers or targets for treating CRC through the in-depth exploration of the molecular mechanism of the interaction among various components in the immune microenvironment in tumorigenesis and development is of great significance.

Normal cells mainly metabolize via mitochondrial oxidative phosphorylation, while most solid tumor cells rely on aerobic glycolysis, a phenomenon known as the Warburg effect [7]. This metabolic reorganization of tumor cells is regulated by various oncogenic proteins and tumor suppressors, including hypoxia-inducible factor (HIF-1α) transcription. It is involved in tumor growth, angiogenesis, and stress-resistant metabolism in the malignant tumor microenvironment (TME) [8]. Glycolytic metabolism can lead to the accumulation of large amounts of lactic acid, which acidifies the TME [9]. Acidosis is acknowledged as the key to malignant progression and somatic evolution. Furthermore, tumor acidosis may be associated with extracellular lactate accumulation and hypoxia [10], contributing to tumor invasion and immune escape. Therefore, lactate has also become one of the hotspots in the current research on TME metabolism. Studies have confirmed [11, 12] that the accumulation of lactic acid in malignant tumors is an important intermediate product of glycolysis and is also involved in regulating cancer development. Tumors with high lactate levels have a high tumor recurrence rate and a high possibility of metastasis, which often leads to poor prognosis for patients [13]. Lactate infiltration in tumors results in immunosuppression, in turn promoting tumor growth [14]. A recent study found that cancer cell-derived lactate promoted breast cancer cells to secrete chemokine C–C Motif Chemokine Ligand 5 (CCL5) through the Notch signaling pathway, which polarized the M2 phenotype of chemotactic macrophages. CCL5 also enhanced survival, aerobic glycolysis, and epithelial-mesenchymal transition (EMT) of breast cancer cells [15]. Furthermore, high concentrations of lactate found in cervical cancer, lung cancer, head and neck tumor, and CRC biopsies were positively associated with the risk of tumor metastasis and predicted poor prognosis in patients with cancer [16].

TME is composed of various cellular and acellular components [17]. Among these, tumor-associated macrophages (TAMs), which account for the largest proportion of cells, can inhibit antitumor immune responses by secreting various mediators, further stimulate angiogenesis, and ultimately promote the occurrence and development of cancer cells [18]. TAMs differentiate into classically activated macrophages (M1 type) and alternatively activated macrophages (M2 type) under different signal stimuli. M1 can inhibit the occurrence and development of tumor cells, while M2 has the opposite effect [19, 20]. Studies have shown that the infiltration of M1 and M2 is directly related to prognosis in patients with CRC [21]. M1 type, represented by markers such as MHCII, CD86, IL-1p, IL-12, IL-23, and iNOS, can induce inflammation, activate a tumor-killing immune response, and ultimately inhibit tumorigenesis. M2 type, represented by markers such as CD206, CD163, IL-10, TGF-β, CCL17, CCL18, CCL22, and Arg-1, mainly inhibits immune inflammatory responses and promotes tumor progression [22]. Many studies demonstrated that the infiltration and distribution of TAMs in cancer tissues were significantly associated with prognosis in patients with cancers, including gastric cancer, breast cancer, bladder cancer, prostate cancer, and kidney cancer [23]. However, the role of TAM in CRC is still controversial. On the one hand, studies have shown that the infiltration of TAM in colorectal tumor tissue is beneficial to the prognosis of patients [24]; on the other hand, TAM promotes proliferation, migration, and invasion of colorectal tumor cells [25]. Many recent studies demonstrated that lactic acid led to CRC progression and poor prognosis, and promoted the M2 polarization of TAM. However, the mechanism of lactate in influencing TAM was rarely explored.

High-mobility group box 1 (HMGB1) is a highly conserved DNA-binding protein ubiquitous in mammalian cells, involved in maintaining nucleosome structure and regulating the transcription of various genes, and has been found abnormally expressed in many human cancers [26]. Intracellularly, HMGB1 is a highly conserved chromosomal protein that can act as a DNA molecular chaperone [27]. Extracellularly, HMGB1 is a typical damage-related molecular pattern, and work together with cytokines, chemokines and growth factors. According to existing research results, HMGB1 regulates various signaling pathways during colorectal cancer development [28]. This study aimed to reveal the regulation mechanism of CRC cell-derived lactate on TAMs and further explore the mechanism underlying lactate accumulation-induced aggravation in CRC.

## Methods

### Cell culture and treatment

SW480 (AW-CCH107), HT29 (AW-CCH054), and THP-1 (AW-CCH098) cell lines were purchased from Abiowell Biotech Co., Ltd. The human CRC cell lines SW480 and HT29 were cultured in Roswell Park Memorial Institute-1640 (RPMI-1640, Gibco), supplemented with 10% fetal bovine serum (WS500T; Ausbian) and 1% penicillin/streptomycin (60162ES; Yeasen Biotech Co., Ltd.). The cells in the normoxia group were incubated in a multi-gas incubator (CHSQ-200-III; Shanghai Chuanhong Experimental Instrument Co., Ltd.) with 20% O_2_, 5% CO_2_, and 75% N_2_ at 37 °C. For hypoxia induction, the cells were incubated under 1% O_2_, 5% CO_2_, and 94% N_2_ for 48 h before starting other experiments.

The THP-1 cells were cultured in the RPMI-1640 medium supplemented with 10% fetal bovine serum, 1% sodium pyruvate (11360070; Thermo Fisher), and 1% penicillin/streptomycin. THP-1 cells were treated with 150 nM phorbol-12-myristate-13-acetate (Sigma–Aldrich) at 37 °C for 48 h to induce the M0 macrophages (M0).

For direct stimulation of M0 cells, the cancer cell supernatant after normoxia or hypoxia incubation was collected by centrifugation at 300* g* for 10 min, followed by centrifugation 2000* g* for 20 min. The supernatant was directly added to the M0 cells.

The indirect contact co-culture was performed in 24-well plates with 8-μm polyethylene terephthalate membrane filters (Corning) separating the lower and upper chambers. THP-1-derived M0 cells were seeded in the upper chamber, and SW480 cells or HT29 cells were seeded in the lower chamber. SW480 cells and HT29 cells were subjected to normoxia or hypoxia treatment before co-culture. After 48 h of incubation, the cancer cells and macrophages were collected for analysis and further experiments.

Further, 3-hydroxy-butyrate acid (3-OBA; Sigma–Aldrich), the G-Protein Coupled Receptor 81 (GPR81) antagonist, was used to inhibit the secretion of lactate in the cancer cells [29]. 3-OBA (3 mM) was added to the cancer cells and incubated for 48 h under normoxic or hypoxic conditions.

For lactate stimulation, gradient concentrations of lactate [30] (purchased from Shanghai Fantai Biotech Co., Ltd., diluted to 0, 5, 10, 20, and 40 mM) were added to the M0 cells and cultured for 6 h. After the incubation of lactate, the macrophages were subjected to other treatments.

Glycyrrhizin (the direct HMGB1 inhibitor, purchased from Sigma–Aldrich) was added to the macrophages at a concentration of 1 nM [31] during incubation with lactate to inhibit the expression of HMGB1 in the macrophages. After the pretreatments, the macrophages were rinsed with phosphate-buffered saline (PBS) and subjected to the co-culture system.

### Western blotting assay

Proteins of xenografts and cells were extracted using Pierce™ IP lysis buffer (87787; Thermal Fisher). 40 μg of the protein was resolved in each well of 10% SDS-PAGE and transferred to Polyvinylidene Fluoride (PVDF) membranes. Then, the blots were blocked by 5% fat free milk and incubated with anti-HIF-1α (1:1000; ab1; Abcam), anti-PGC1α (1:1000; ab106814; Abcam), anti-MCT1 (1:3000; ab90582; Abcam), anti-MCT4 (1:1000; ab234728; Abcam), anti-CD174 (1:2000; ab235831; Abcam), anti-HMGB1 (1:1000; ab18256; Abcam), anti-E-cadherin (1: 20000; ab40772; Abcam), anti-N-cadherin (1:10000; ab76011; Abcam), anti-Bcl-2 (1:1000; ab32124; Abcam), anti-p-GSK (1:10000; ab75814; Abcam), anti-GSK (1:8000; ab32391; Abcam), anti-ERK (1:10000; ab184699; Abcam), anti-p-ERK (1:1000; ab201015; Abcam), anti-JNK (1:1000; ab179461; Abcam), anti-p-JNK (1:5000; ab124956; Abcam), anti-p38 (1:5000; ab170099; Abcam), anti-p-p38 (1:1000; ab178867; Abcam) primary antibodies at 4 °C overnight. The day after, rabbit anti mouse IgG secondary antibody (1:2000; ab6728; Abcam) was incubated with the membranes at room temperature for 2 h. GAPDH was used as control. Finally, protein bands were visualized using the Ultra High Sensitivity ECL Substrate Kit (ab133409; Abcam).

### Wound healing assay

SW480 and HT29 cells were inoculated into a six-well plate and cultured at 37 °C in the presence of 5% CO_2_ until the cell confluence reached 90%. Then, a line was scraped into the monolayer cells using the tip of a micropipette. PBS was used to rinse the plate twice to remove the nonadherent cells and cultured in a serum-free RPMI-1640 for 24 h. The images were taken and observed after 0, 24, and 48 h under a microscope, and the wound healing rates were analyzed using Image J software (version 1.8.0).

### Colony formation assay

100 μl cells suspension (1 × 10^4^ cells/ml) per well was injected into a 6-well plate, and they were cultured in dulbecco’s modified eagle medium (DMEM) containing 20% FBS for 10 days. Then, these cells were rinsed twice with PBS, fixed with 4% paraformaldehyde (Labcoms) and stained with 0.1% crystal violet. The cell clusters containing > 50 cells were counted.

### Enzyme-linked immunosorbent assay (ELISA)

ELISA kit (K607-100, BioVision) was used to measure the level of lactate in the cell supernatants of SW480 and HT29 cells obtained through centrifugation according to the manufacturer’s protocols.

### Real-time quantitative polymerase chain reaction (RT-qPCR)

Total RNA of tumor tissues and the cells were extracted by MolPure^®^ Cell RNA Kit (Yeasen Biotechnology Co., Ltd.). Reverse transcription and PCR amplification were carried out using Quant One Step qRT-PCR Kit (Probe) (LM-0102; LMai Biotech Co., Ltd.). All primers were synthesized by Tsingke Biotech Ltd., GAPDH was used as internal reference for mRNAs. Fold changes of the RNAs were assessed using 2^−ΔΔCt^ method. The sequences of the primers used were listed in Table 1.

**Table 1:** Sequences of the primers used in RT-qPCR

Gene	Sequence (5′- > 3′)
MCT1	Forward AGGTCCAGTTGGATACACCCC Reverse GCATAAGAGAAGCCGATGGAAAT
MCT4	Forward AGGTATCCTTGAGACGGTCAG Reverse CAAGCAGGTTAGTGATGCCG
PGC1α	Forward TCTGAGTCTGTATGGAGTGACAT Reverse CCAAGTCGTTCACATCTAGTTCA
CD147	Forward GAAGTCGTCAGAACACATCAACG Reverse TTCCGGCGCTTCTCGTAGA
HMGB1	Forward TATGGCAAAAGCGGACAAGG Reverse CTTCGCAACATCACCAATGGA
GAPDH	Forward CTGGGCTACACTGAGCACC Reverse AAGTGGTCGTTGAGGGCAATG

### Flow cytometry assay

Cell apoptosis was examined by Annexin V-FITC/PI Apoptosis Detection Kit (40302ES20, Yeasen Biotech Co., Ltd.). Macrophages were trypsinized and rinsed with pre-chilled PBS twice. Then, 1 × 10^5^ cells were collected and resuspended in 100 μl binding buffer. 5 μl of Annexin V-FITC was ulteriorly added into the cells and incubated for 15 min under room temperature in dark, followed by 5 μl of PI staining solution. The apoptotic cells were analyzed on FACSCalibur flow cytometer (BD Biosciences).

For assessing the polarization of M0 cells, the single macrophages were incubated with anti- MHCII (1:1000, ab20181, Abcam), anti-CD86 (1:1000, ab239075, Abcam), anti-CD163 (1:1000, ab182422, Abcam), and anti-CD206 (1:1000, ab270647, Abcam) for 40 min, and then rinsed with PBS. Next, the cells were stained with PC5.5‐conjugated 7AAD (Biolegend) for 5 min to remove dead cells.

### Transwell migration assay

SW480 and HT29 cells were resuspended at the density of 2 × 10^5^ cells/Ml in serum-free RPMI-1640 medium and starved for 24 h. Transwell chambers (3422; Corning) were inserted into a 24-well plate. The cell suspension was added to the upper Transwell chamber, and 600 μL of RPMI-1640 with 20% FBS was added to the lower chamber. After incubation for 24 h, the cells remaining in the upper chamber were gently removed, and the cells moving to the lower chamber were fixed with methanol and stained using 0.1% crystal violet. The stained cells were observed and counted under a light microscope (XSP-1200A; Shanghai CSOIF Co., Ltd.) at the magnification of 200 ×. Five visual fields of each well were randomly chosen.

### Tumorigenicity assay

Humanized BALB/c male nude mice (6 weeks old; weight: 21.2 ± 0.8 g) were purchased from Cyagen Biosciences. The mice were raised under the SPF condition with a 12 h dark/light cycle. After keeping the mice in the laboratory for a week, xenografts were induced by subcutaneous injection of 10^7^ SW480 cells. After inoculation, 3-OBA (100 µM) was orthotopically injected into the mice at a dose of 2 µL every 5 days, or HMGB1 inhibitor [RAGE antagonist peptide (RAP, 10 µM/kg) and ethyl pyruvate (EP, 1 mM/kg), purchased from Sigma–Aldrich] was injected into the mice at a 3 day interval. The tumor volumes were measured every week using the following formula: *V* = *ab*^2^/2, where *a* is the long diameter and *b* is the short diameter. The mice were sacrificed using 150 mg/kg pentobarbital i.v. after 30 days of modeling. The tumors were collected, weighed, and embedded for further use.

### Hematoxylin and eosin (H&E) staining

Paraffin sections of the tumor tissues were administrated to H&E staining assay to assess tissue damage degree. The sections were stained with hematoxylin for 5 min and counterstained by eosin for 3 min. Five fields of the stained slide were randomly captured by an optical microscope.

### Immunofluorescence (IF)

Macrophages were fixed with 4% paraformaldehyde, permeabilized with 0.1% Triton X-100 for 20 min and blocked with 3% BSA. Next, the cells were incubated with the primary anti-HMGB1 (1:1000; 20R-FR012; Fitzgerald) antibody at 4 °C overnight. Then, the cells were incubation with secondary goat anti-rat IgG-iFluor 488 antibodies (CM008-10A2F; Chamot Biotech Co., Ltd.) at 37 °C for 1 h, and counter-stained with DAPI for 15 min. ZEISS LSM 9 laser confocal microscopy was used to observe the fluorescently-labeled cells.

### Isolation of TAMs

After resection, the xenografts were immersed in RPMI-1640 and minced into 0.1 mm^3^ cubes. Type II collagenase (1 mg/mL), hyaluronidase (0.2 mg/mL), and DNAse I (0.2 mg/mL) were added to the medium to digest the minced tissues at 37 °C for 1 h. The cell suspension was filtered through a filter with a pore size of 40 μm and centrifuged at 800* g* for 5 min. Then, the cell suspension was centrifuged with Percoll (Amersham). After gradient separation, the cells in the lower layer were subjected to flow cytometry for identifying the macrophages.

### Ki67 staining

The sections of xenografts were previously dewaxed and rehydrated. After antigen retrieval, the sections were blocked with goat serum for 20 min at room temperature. Anti-Ki67 (1:2000; ab15580) primary antibody was incubated with the sections at room temperature for 2 h followed by goat anti-rabbit IgG H&L (HRP) secondary antibody (1:1000; ab6721). DAB developing kit (ab64238; all from Abcam) and hematoxylin were used to develop the sections.

### Statistical analysis

Each experiment was performed in triplicate. The data were analyzed using GraphPad Prism (version 9.1.1.225, GraphPad Software Inc.). The data were presented as mean ± standard deviation. The Student *t* test was performed for the two-group comparison, and analysis of variance was used for comparing multiple groups, followed by Duncan’s post hoc test. A *P* value < 0.05 indicated a statistically significant difference.

## Results

### Hypoxia-treated cancer cells exhibited increased mobility and M2 polarization of THP-1 cells

**Fig. 1:**
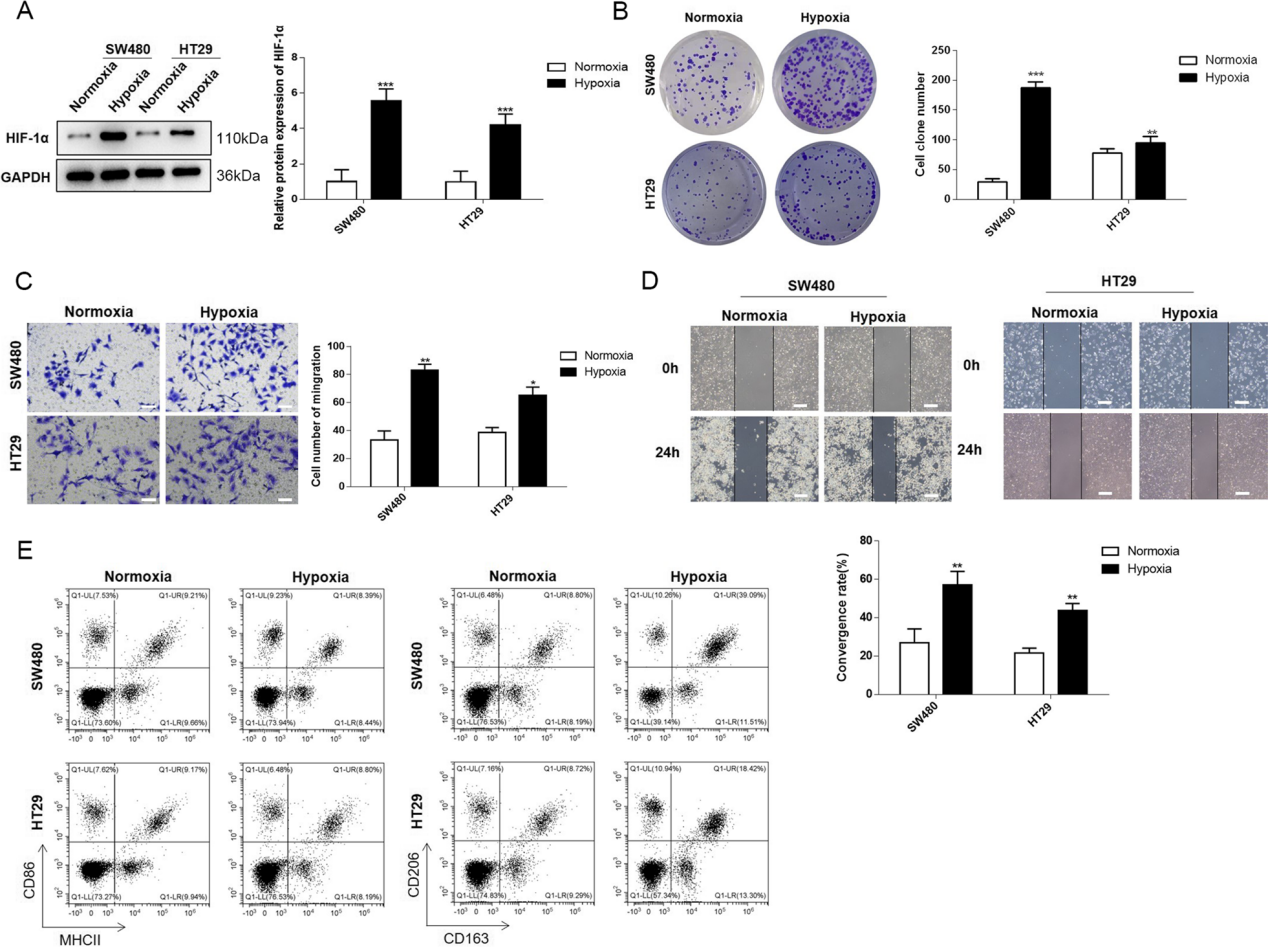
Hypoxia treated cancer cells exhibited increased mobility and promoted M2 polarization of THP-1. **A** HIF-α levels were detected in SW480 and HT29 cells under normoxia and hypoxia conditions using western blotting assay. **B** Colony formation, **C** Transwell cell migration and **D** wound healing assays were performed to assess cell mobility. **E** M1 and M2 phenotypes of macrophages were examined by flow cytometry assay. CD86 and MHCII were indicators for M1, CD163 and CD206 were indicators for M2. Data were presented as mean ± SD and each assay was performed for three times. ^*^P < 0.05; ^**^P < 0.01; ^***^P < 0.001

First, SW480 and HT29 cell lines were cultured under hypoxic conditions to mimic the hypoxia TME in vitro. After a 2 day-culturing under normoxic or hypoxic conditions, the HIF-α level was determined by western blot assay to assess whether the hypoxia stress was successfully induced into the cancer cells. The results showed that the expression of HIF-1α was significantly promoted under hypoxic conditions (Fig. 1A), suggesting the successful establishment of the hypoxia model. Next, we evaluated the cell growth and metastasis abilities of the cancer cells. The colony formation results indicated that both SW480 and HT29 cell lines showed notably elevated colony numbers after a 2 day culturing under hypoxic conditions compared with normoxic conditions (Fig. 1B). Transwell migration assay was performed, and the results demonstrated that the hypoxic conditions prominently enhanced the migration of SW480 and HT29 cells (Fig. 1C). Similarly, the wound healing rates of SW480 and HT29 cell lines were significantly increased after hypoxic treatment (Fig. 1D). Subsequently, the cell supernatant was collected from the cancer cells after modeling to stimulate the THP-1-derived macrophages. When the macrophages were exposed to the supernatant from hypoxic groups, no obvious change was observed in the cell number of M1 macrophages. However, the cell number of M2 macrophages was observably enhanced (Fig. 1E). These results consistently indicated that hypoxic conditions contributed to the malignancy of CRC cells, and the cell supernatant under hypoxic conditions could promote M2 polarization of macrophages.

### Hypoxic conditions induced lactate secretion from cancer cells, further facilitating M2 polarization of macrophages

**Fig. 2:**
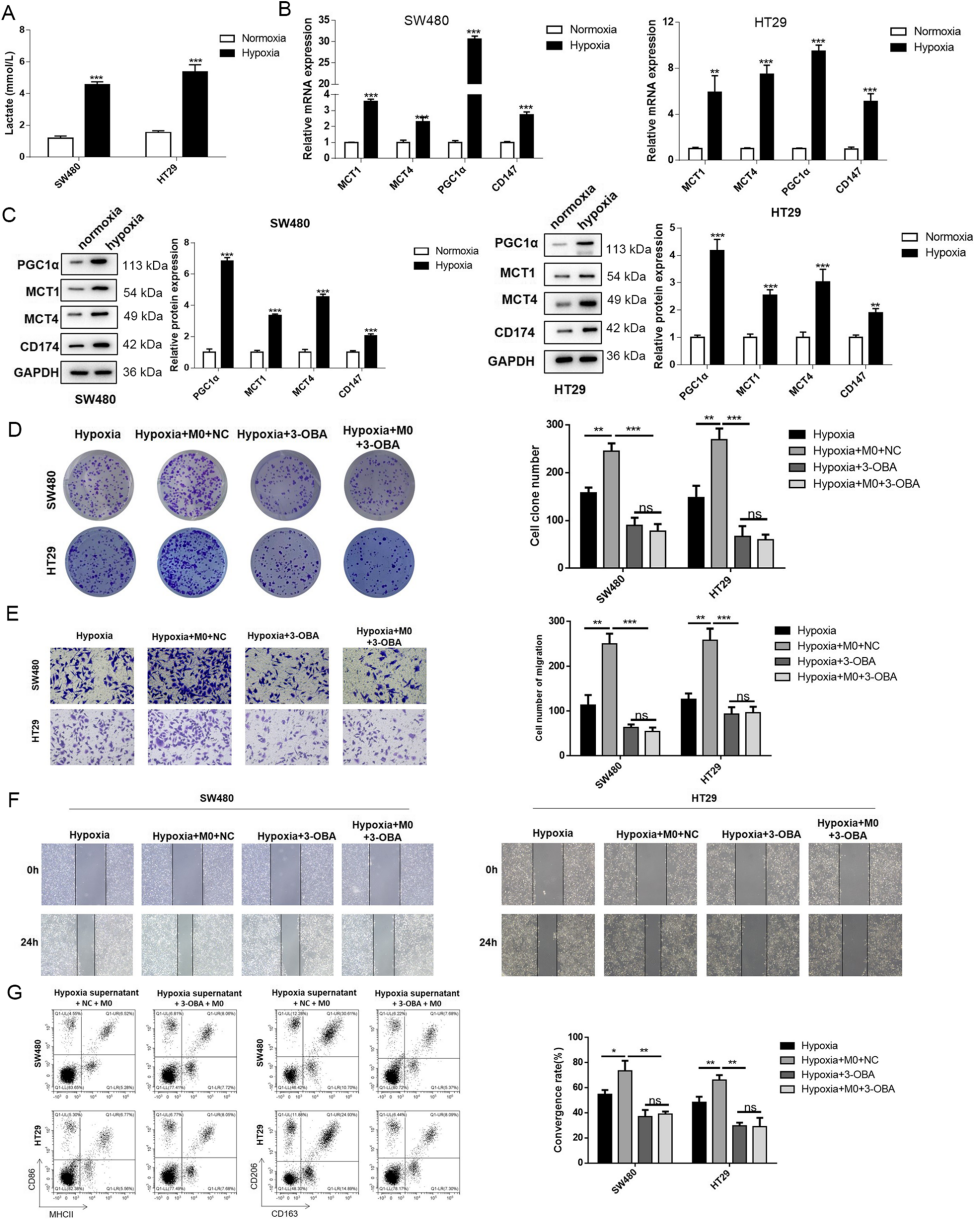
Hypoxia condition induced lactate secretion of cancer cells, further facilitated M2 polarization of macrophages. **A** Lactate contents in the supernatant of SW480 and HT29 cells were measured by ELISA kit. **B** mRNA levels and **C** protein levels of lactate-related genes were detected by RT-qPCR. **D** Colony formation, **E** Transwell cell migration and **F** wound healing assays were performed to assess cell mobility. **G** M1 and M2 phenotypes of macrophages were examined by flow cytometry assay. CD86 and MHCII were indicators for M1, CD163 and CD206 were indicators for M2. Data were presented as mean ± SD and each assay was performed for three times. ^*^P < 0.05; ^**^P < 0.01; ^***^P < 0.001

Active glycolysis in the cancer cells was widely acknowledged, and lactate, as the main metabolite of glycolysis, was found to influence the TME, including TAMs. Therefore, we determined the lactate level in the cell supernatant of hypoxic cancer cells. The ELISA results showed that the lactate levels were dramatically increased in the supernatant of hypoxic cancer cells (Fig. 2A). Then, the expression of genes related to lactate transport and metabolism was analyzed. The mRNA levels of PGC1α, MCT1, MCT4, and CD147 were all significantly promoted in the hypoxic cancer cells (Fig. 2B). Besides, the protein expression of these lactate-related genes exhibited the same trend as the mRNA levels (Fig. 2C). These results showed that lactate was significantly activated in the cancer cells under hypoxic conditions. To further verify this result, 3-OBA was added to inhibit lactate secretion in the cancer cells. In the hypoxia group, the co-culture with macrophages notably increased the colony numbers of SW480 and HT29 cells. However, in the 3-OBA-treated group, the colony formation was inhibited; even co-culture with macrophages showed no increase in the colony numbers (Fig. 2D). The results of Transwell and wound healing assays showed that 3-OBA also suppressed the migration capacity of the cancer cells, and the co-culture with macrophages after 3-OBA treatment did not cause evidently increased migration capacity in the cancer cells (Fig. 2E, F). Moreover, 3-OBA inhibited M2 polarization of the macrophages in the co-culture system (Fig. 2G). These results elucidated that lactate secreted by hypoxic cancer cells could accelerate the M2 polarization of macrophages, and the M2-type macrophages in turn contributed to the carcinogenicity of cancer cells.

### Cancer cell-derived lactate upregulated HMGB1 in macrophages, and HMGB1 further promoted carcinogenic behaviors in cancer cells

**Fig. 3:**
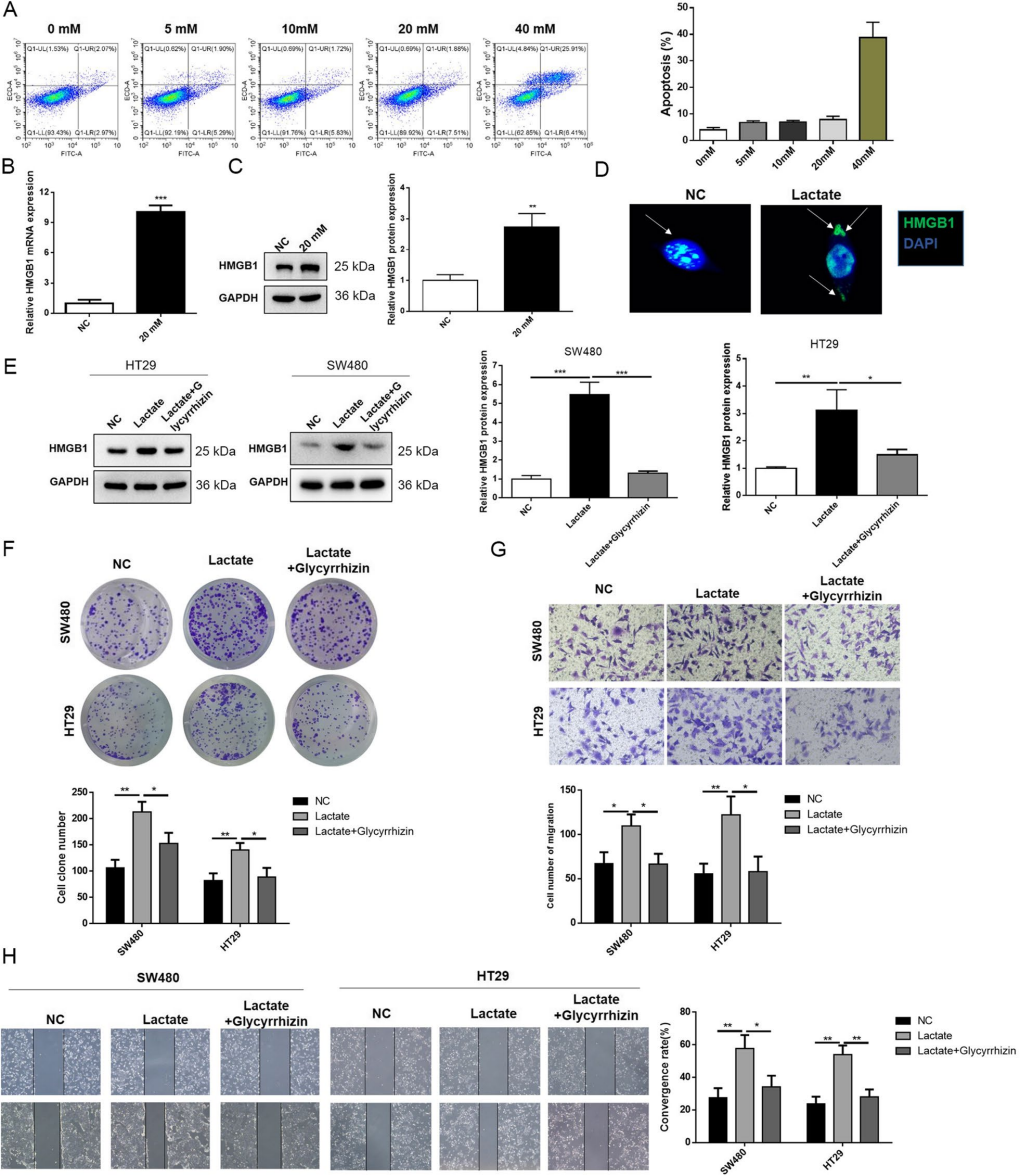
Cancer cell-derived lactate upregulated HMGB1 in macrophages and HMGB1 further promoted carcinogenic behaviors in cancer cells. **A** Cell apoptosis of macrophages was evaluated by flow cytometry. **B** mRNA and **C** protein levels of HMGB1 in macrophages were determined. **D** The location of intracellular and extracellular HMGB1 was visualized using immunofluorescence. **E** HMGB1 protein expressions were detected in SW480 and HT29 cells by western blotting. **F** Colony formation, **G** Transwell cell migration and **H** wound healing assays were performed to assess cell mobility. Data were presented as mean ± SD and each assay was performed for three times. ^*^P < 0.05; ^**^P < 0.01; ^***^P < 0.001

Lactate was added to the macrophages to further explore how macrophages exerted tumor-promoting effects on cancer cells. Based on previous findings [30], the gradient concentration of lactate was set as 0, 5, 10, 20, and 40 mM to determine an optimal concentration of lactate for the following experiments. After culturing the macrophages with different levels of lactate for 6 h, the cell death of macrophages was evaluated. The results indicated that when the lactate concentration reached 40 mM, the apoptosis rate of macrophages sharply increased. Hence, the lactate concentration of 20 mM was selected to conduct the following experiments (Fig. 3A). A previous report revealed the impact of lactate on HMGB1 in the macrophages in sepsis [32]. In this study, we intended to confirm the relationship in CRC. The mRNA and protein levels of HMGB1 in the lactate-induced macrophages were prominently elevated (Fig. 3B, C). In addition, the images of IF assay showed that lactate treatment promoted the extracellular secretion of HMGB1 by macrophages (Fig. 3D). The levels of HMGB1 were determined in the cancer cells after co-culture with macrophages to further investigate whether the macrophage-derived HMGB1 was taken up by cancer cells to promote cancer progression. Glycyrrhizin (1 nM) was added to the macrophages to inhibit HMGB1 expression [31]. The level of HMGB1 in cancer cells was increased by lactate-treated macrophages, while the increase was inhibited by glycyrrhizin (Fig. 3E). Lactate-treated macrophages significantly enhanced the growth of SW480 and HT29 cell lines; however, glycyrrhizin reversed the effects of lactate inducement (Fig. 3F). Additionally, the cell migration of the two cancer cell lines was accelerated after co-culture with lactate-induced macrophages, but glycyrrhizin abrogated the effect of lactate (Fig. 3G, H). These results indicated that macrophages enhanced cancer cell mobility through lactate-induced HMGB1 upregulation.

### HMGB1 promoted the malignancy of cancer cells by activating EMT, Wnt, and ERK signaling pathways

**Fig. 4:**
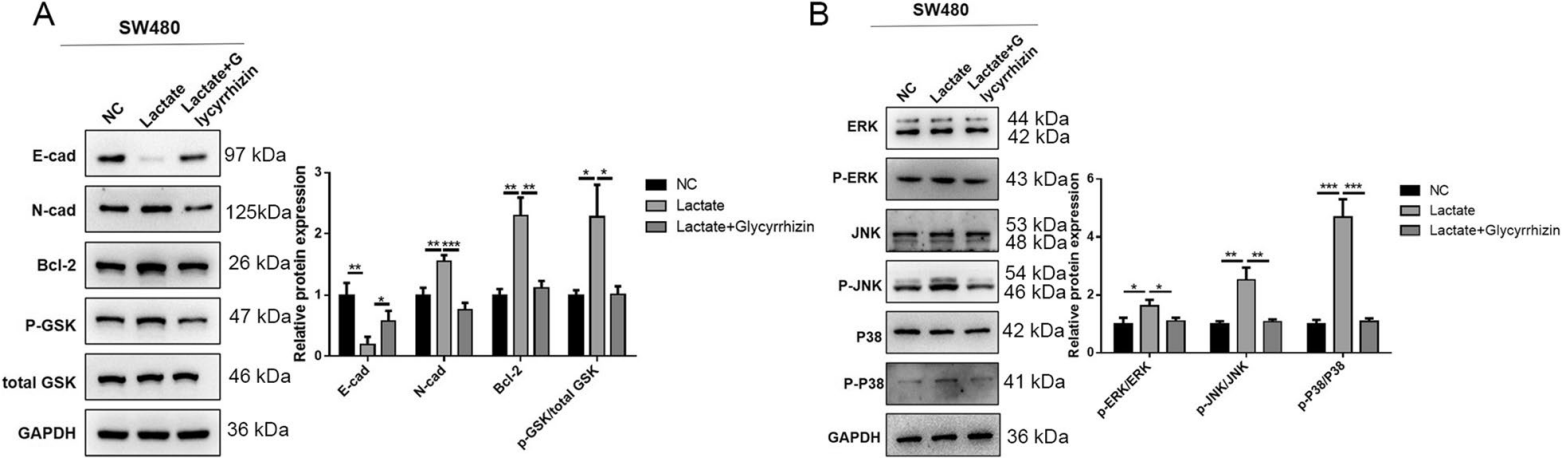
HMGB1 promoted malignancy of cancer cell through activating EMT process, and Wnt and ERK signaling. **A**, **B** The expressions of EMT process, and Wnt and ERK signaling were detected using western blot assay in SW480 cells. Data were presented as mean ± SD and each assay was performed for three times. ^*^P < 0.05; ^**^P < 0.01; ^***^P < 0.001

The common signaling pathways involved in cancer progression were examined. Previous studies confirmed that HMGB1 could upregulate the ERK1/2 pathway in CRC, and Wnt and ERK signaling pathways were activated by HMGB1 in pancreatic cancer [27, 33]. We examined the previous findings in the present study. After the co-culture with macrophages underwent lactate treatment, the level of E-cadherin decreased, while the levels of N-cadherin and Bcl-2 and the ratio of p-GSK/total GSK were all enhanced in the cancer cells. Glycyrrhizin reversed the progress of EMT (Fig. 4A). Similarly, the activation rates of ERK, JNK, and p38 significantly increased, but glycyrrhizin partially restored the activation of ERK compared with that in the control group (Fig. 4B). These results further proved the carcinogenic effects of HMGB1 on the molecular level.

### Inhibition of lactate or HMGB1 played a similar anti-tumor role in the tumor-bearing mice

**Fig. 5:**
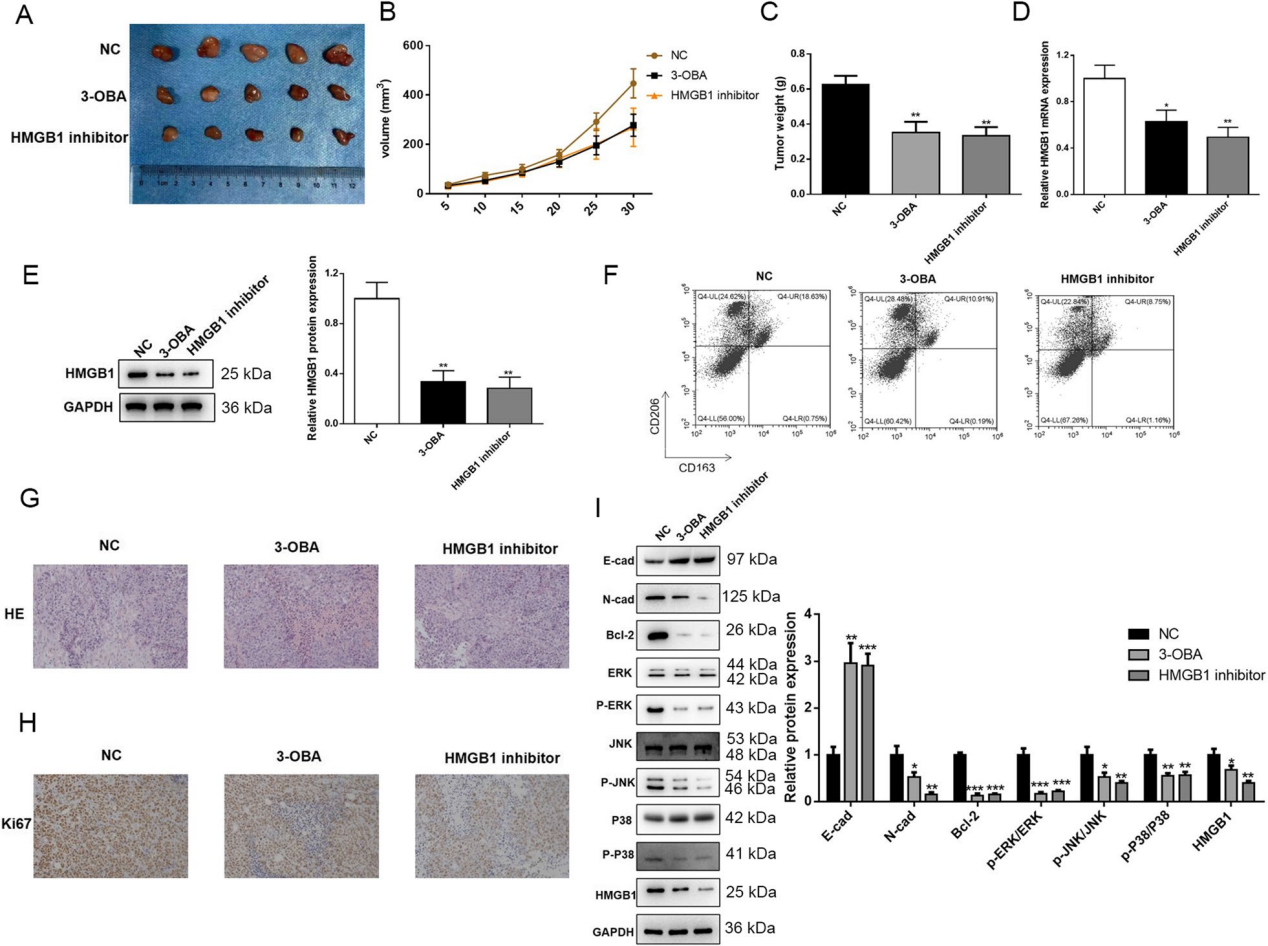
Inhibition of lactate or HMGB1 showed similar anti-tumor role in the tumor-bearing mice. **A** After modeling, the xenografts were resected and photographed. **B** Tumor volumes were documented every 5 days. **C** Tumor weights were measured after 30 days of modeling. **D** mRNA and **E** protein levels of HMGB1 were evaluated in the mice. **F** M2 polarization was assessed in the tumor-associated macrophages using flow cytometry. **G** HE and **H** Ki67 staining assays were carried out to determine the malignancy of tumors. **I** The expressions of EMT process, ERK signaling and HMGB1 were detected by western blot assay. CD163 and CD206 were indicators for M2. Data were presented as mean ± SD and each assay was performed for three times. ^*^P < 0.05; ^**^P < 0.01; ^***^P < 0.001

Based on the aforementioned results, the SW480 cell line was more sensitive to different treatments. Therefore, SW480 cells were selected to induce ectopic xenografts in nude mice. As shown in Fig. 5A, B, the tumor volume significantly decreased in the mice injected with 3-OBA or HMGB1 inhibitor (RAP + EP) compared with the control group. The tumors were resected and weighed after 30 days of modeling. The tumor weights in the 3-OBA and HMGB1 groups were prominently lower than those in the control group (Fig. 5C). Further, TAMs were isolated from the tumors, and the polarization state of TAMs was analyzed. The results indicated that 3-OBA and HMGB1 inhibitor both suppressed M2 polarization of the TAMs (Fig. 5F), and the mRNA and proteins levels of HMGB1 were reduced (Fig. 5D, E). The H&E staining images showed that the infiltration of lymphocytes and macrophages was more obvious in the 3-OBA and HMGB1 inhibitor groups (Fig. 5G). Also, the number of Ki67-positive cells were observably reduced in the 3-OBA and HMGB1 inhibitor groups (Fig. 5H). Furthermore, the activations of EMT process and ERK signaling were suppressed by 3-OBA and HMGB1 inhibitor (Fig. 5I).

## Discussion

Cancer cell-derived lactate is a common metabolite in the TME. The acidic TME caused by the accumulation of lactate further promotes cancer progression. TAM is a major cellular component in TME, and cancer cells suppress inflammation and immune cell infiltration by promoting M2 polarization of macrophages. These factors greatly hinder the treatment effects and prognosis of CRC. The present study revealed that lactate produced by cancer cells enhanced the M2-type ratio of TAMs and elevated the level of HMGB1 in TAMs. The upregulated HMGB1 was secreted into the extracellular environment, further increasing the level of HMGB1 in the cancer cells. HMGB1 activated the ERK, Wnt, and EMT signaling pathways to aggravate the malignant behaviors of CRC cells. Our study preliminarily explored the interaction between TAMs and cancer cells based on HMGB1 in CRC.

The relationship between macrophages, tumor cells, and other components of the TME is dynamic and heterogeneous [34]. Studies found extensive interactions between TAMs and TME. The properties of TAMs depend on the stimulation of various components in TME to a large extent; TAMs can affect TME, thereby promoting tumor progression [35]. TAMs originate from mononuclear precursors, and the effect of chemokines, such as CSF-1, CCL2, VEGF, and TGF-β, is required for monocytes to reach the tumor site in the colon [35]. In TME of CRC, the production of cytokines, growth factors, and metabolites by cancer and immune cells promotes the pre-tumor polarization of TAMs. Studies showed that lactic acid produced by the enhanced glycolytic activity of cancer cells caused TME acidification. The acidified TME induced regulatory macrophages through G protein–coupled receptors and IL-1β-converting enzymes, enabling the production of VEGF and arginase [36]. The increased expression by TAMs induced the M2 phenotype of TAMs [37]. Lian et al. [38] co-cultured colon cancer cell lines (HCT8 or HCT116) with human myeloid leukemia mononuclear cells (THP-1). They found that the levels of IL-10 and arginase-1 increased, indicating that the colon cancer cells were involved in the M2 polarization of THP-1, which was also confirmed by using the EGFR antibody mAb225 and the PI3K inhibitor LY294002. In this study, hypoxia-induced cancer cells were used to simulate the in vivo cancer environment in in vitro experiments, and the results were the same as the aforementioned findings; macrophages tended toward M2 polarization under the induction of CRC cells.

Studies reported that the activation of ERK/STAT3 was an essential link in the lactate signaling pathway. Inhibiting the ERK/STAT3 signaling pathway could inhibit the growth of breast cancer and the formation of a large number of blood vessels and eliminating the lactate-induced polarization of M2 macrophages [39]. The study demonstrated that lactic acid could inhibit the activity of lymphocytes and participate in tumor formation. In CRC, Wang et al. [40] proved that PTTG3P was abnormally upregulated in patients with CRC, and the upregulation of PTTG3P promoted cell growth and lactate accumulation by YAP1, to further polarize TAMs into M2 type. In addition, Tu et al. [41] showed that the lactate produced by CRC cells enhanced ROS accumulation in macrophages to activate the NLRP3 inflammasome. Lactate could also induce TGF-β in the TME, in turn inhibiting inflammasome activation induced by lactate and other canonical ligands in macrophages. Our study found that the lactate produced by CRC cells was responsible for the M2 polarization of TAMs, which led to an anti-inflammatory TME similar to previous findings.

As the main components of TME, TAMs play an important role in CRC invasion and migration. Lan et al. [25] showed that M2-type TAM-derived exosomes induced the migration and invasion of CRC cells and exosomes highly expressed miR-21-5p and miR-155-5p, which were transferred to miR-155-5p through exosomes. CRC cells bind to the BRG1-coding sequence to target and downregulate the expression of BRG1, thereby promoting the migration and invasion of colon cancer cells. In addition, Lim et al. [42] found that TAMs induced higher calcium-binding protein S100A8/A9 mRNA expression levels in colon cancer TME in an ERK-dependent manner than in normal tissues; also, they could stimulate cancer cell migration. EMT is frequently activated during tumor invasion and metastasis, in which TAMs play a key role. Wei et al. [43] found that TAMs could enhance the invasion and metastasis of colon cancer cells by secreting IL-6 and regulating the JAK2/STAT3/miR5063P/FXQ1 axis to induce the EMT program. Similarly, we found that lactate-treated TAMs could significantly enhance the malignancy of CRC cells.

The expression of HMGB1 in CRC tissue is significantly increased, and the expression of HMGB1 is directly involved in the infiltration and migration of CRC cells; the higher the expression level of HMGB1, the later stage of CRC [28]. HMGB1 is an essential member of the HMG family. It is highly conserved in the nucleus, and is mainly involved in DNA repair, transcription, and genome stability. Further, it promotes inflammation, immunity, cell proliferation, autophagy, apoptosis, and metastasis after epigenetic inheritance outside the nucleus [44]. The increase in the extracellular release of HMGB1 can inhibit the antitumor effect of radiotherapy [45]. HMGB1 plays an important role in promoting CRC cell proliferation, invasion, and migration. Studies showed that silencing HMGB1 expression could significantly inhibit CRC cells. On the one hand, it might be related to the activation of the JAK/STAT3 pathway; on the other hand, it might be associated with the production and phosphorylation of proteins related to the RAGE signaling pathway. When the expression of cleaved caspase-3 and PCNA increased, the expression of HMGB1 also increased accordingly. Moreover, the antisense oligodeoxynucleotides synthesized by HMGB1 also reduced the phosphorylation of ERK1/2, Rac1, and protein kinase B and the production of iNOS, NF-κB, and matrix metalloproteinase 9 [46]. The researches on HMGB1 have mainly focused on cancer cells for now. In the present study, we were inspired by a recent report elucidating that HMGB1 accumulated in macrophages and was secreted into the extracellular environment in sepsis [32]. We confirmed that HMGB1 accumulated under the inducement of CRC cell-derived lactate and observed that HMGB1 was also released into the extracellular environment, verifying the finding in CRC.

Crosstalk frequently occurs between TAMs and cancer cells in TME. For example, Zhao et al. [47] elucidated that miR-934 was notably upregulated in CRC, and exosomes derived from CRC cells could carry miR-934 to the TAMs to inhibit PTEN expression and activate PI3K/Akt signaling, resulting in the M2 polarization of TAMs. Interestingly, the M2 macrophages further secreted CXCL13 to induce CRC liver metastasis, and CXCL13 acted as a positive regulator of the CXCR5/NF-κB/p65/miR-934 loop. Yang et al. demonstrated that [48] CRC cells underwent EMT, enhancing the level of miR-106b in TAMs; also, miR-106b inhibited PDCD4 to activate PI3Kγ/Akt/mTOR signaling to accelerate M2 polarization. In turn, the M2 macrophages promoted EMT progression in CRC cells. The present study found that the upregulated HMGB1 in macrophages in turn increased the level of HMGB1 in CRC cells, and ERK1/2, Wnt, and EMT signaling pathways were activated, further enhancing the malignancy of CRC cells. This positive feedback between TAMs and cancer cells was rarely investigated in CRC. Our findings laid the foundation for future works to improve the prognosis of CRC. Despite the current results, the mechanism of HMGB1 interchange between tumor cells and tumor-associated macrophages is still unclear. We will further investigate the mechanism in our future work.

## Conclusions

In summary, the present study revealed that lactate produced by CRC cells could stimulate M2 polarization and upregulate HMGB1 of TAMs, which in turn enhanced the level of HMGB1 in CRC cells to aggravate carcinogenic behaviors. However, the delivery method of HMGB1 from TAMs to cancer cells is still unknown, and the downstream regulatory mechanism needs to be further explored. In our future studies, we will focus on these points to more completely comprehend the crosstalk in CRC.

## Data Availability

Not applicable.

## Abbreviations

CRC: Colorectal cancer
TAMs: Tumor-associated macrophages
TME: Tumor microenvironment
HIF-1α: Hypoxia-inducible factor-1α
H&E: Hematoxylin–eosin staining
IF: Immunofluorescence
IHC: Immunohistochemistry
3-hydroxy-butyrate: 3-OBA
CCL5: C–C motif chemokine ligand 5
EMT: Epithelial-mesenchymal transition
RPMI-1640: Roswell park memorial institute-1640
GPR81: G-protein coupled receptor 81
PVDF: Polyvinylidene fluoride
DMEM: Dulbecco’s modified eagle medium
ELISA: Enzyme-linked immunosorbent assay
